# Genetic progress in rice yield: preliminary insights from historically released varieties in Sub-Saharan Africa

**DOI:** 10.3389/fpls.2025.1670651

**Published:** 2026-01-21

**Authors:** Shailesh Yadav, Vimal Kumar Semwal, Gbènakpon Aubin Y. G. Amagnide, Esther Pegalepo, Kora Orou Kobi, Kossi Lorimpo Adjah, Nana Kofi Abaka Amoah, Raafat El-Namaky, Negussie Zenna, Epa N’da Ghislain Noumouha, Faye Omar Ndaw, Muhammad Liman Muhammad, Laho Mamadou Barry, Medoune Khouma, Dolo Menidiou, Dule Zhao, Baboucarr Manneh

**Affiliations:** 1 Genetic Diversity and Improvement (GDI), Africa Rice Center (AfricaRice), Bouaké, Côte d’Ivoire; 2 Genetic Diversity and Improvement (GDI), Nigeria Country Office, Africa Rice Center (AfricaRice), Ibadan, Nigeria; 3 Africa Rice Center (AfricaRice), Saint-Louis, Senegal; 4 Madagascar Country Office, Africa Rice Center (AfricaRice), Antananarivo, Madagascar; 5 National Center for Agronomic Research (CNRA), Man, Côte d’Ivoire; 6 Senegalese Agricultural Research Institute (ISRA), Dakar, Senegal; 7 National Cereals Research Institute (NCRI), Badeggi, Nigeria; 8 Guinea Agricultural Research Institute (IRAG), Conakry, Guinea; 9 Institut d’Economie Rurale (IER)/Regional Center for Agronomic Research (CR&A)-Sikasso, Sikasso, Mali

**Keywords:** ERA trials, genetic trends, grain yield, rice, sub-Saharan Africa

## Abstract

Rice is a vital staple crop in sub-Saharan Africa (SSA), where improving grain yield is critical for food security and economic growth. Assessing genetic gain over time is essential for measuring breeding effectiveness and guiding future strategies and evaluating the return on investment in rice improvement programs. This study aimed to estimate baseline genetic gains in grain yield from AfricaRice-bred released and pre-released varieties between 1986 and 2020 using multilocation data from ERA trials. “ERA trials” are designed to estimate historic rates of genetic gain for grain yield by testing a series of varieties released over different years or different breeding periods or “eras.” Three sets of trials representing irrigated lowland, rainfed lowland, and rainfed upland ecologies were conducted in 2021 and 2022 at AfricaRice breeding stations in Côte d’Ivoire, Nigeria, and Senegal as well as National Agricultural Research and Evaluation Systems (NARES) sites in Burkina Faso, Guinea Conakry, and Mali. The trials were conducted using an alpha- lattice design with three replications, and data were collected on grain yield, plant height, and days to flowering. A two-stage analysis was implemented, where genotype-by-environment (G × E) means from the first stage were used in the second stage to model G × E interaction with a second-order factor analytic model, thereby accommodating genetic heteroscedasticity across environments and enabling estimation of genetic trends. Finlay–Wilkinson regression model identified high-performing and stable varieties in each ecology. Consistent positive genetic trends were estimated across all the ecologies, though gains remained low: 12 kg/ha/year (0.34%) in rainfed lowland, 10 kg/ha/year (0.27%) in rainfed upland, and 7 kg/ha/year (0.14%) in irrigated lowland. The top-performing varieties contributed maximum gains was ARICA 18 in rainfed lowland (16 kg/ha/year), FARO 59 (NERICA 8) in rainfed upland (11 kg/ha/year), and Yiriwamalo in irrigated lowland (8 kg/ha/year).These results highlight the steady progress of AfricaRice breeding programs and underscore the need for continued investment in rapid varietal development using modernized breeding tools to deliver high-yielding, climate-resilient, and market-driven rice varieties for SSA.

## 1 Introduction

Rice (*Oryza sativa* L.) has emerged as a strategic food and cash crop in Sub-Saharan Africa (SSA), driven by rapid urbanization, population growth (projected to double by 2050), and shifting consumer preferences (Arouna et al., 2021). Annual rice consumption in SSA is growing at 6%, the fastest rate globally, with per capita intake expected to reach 30 kg/year by 2030, second only to Asia (Rahman et al., 2023). Rice now provides 20-25% of daily calories for over 500 million people in SSA, surpassing traditional staples like cassava and sorghum in urban markets (FAOSTAT, 2023). However, domestic rice production in SSA meets only 60% of demand, necessitating 14–15 million tons of annual imports at a cost exceeding US$6 billion, a drain on foreign reserves and a vulnerability to global price shocks (World Bank, 2022).

Rice in SSA is cultivated across diverse agroecological systems, each having unique challenges and yield potential. Rainfed lowland areas, which account for 40% of the total rice-growing area, are prone to drought and flood, confining yields to an average of 1.5-2.5 tons/ha (Diagne et al., 2013). Rainfed upland systems cover approximately 35% of the area and are typically grown on level or sloping, unbounded fields. Under rainfed upland, yields are even lower, often not exceeding 1.2 tons/ha, due to recurrent drought, poor soil fertility, and intense weed pressure (Saito et al., 2018). Irrigated lowland ecologies, representing 21% of the rice area, have the highest yield potential (4–6 tons/ha), though their productivity is often compromised by abiotic stresses such as salinity and iron toxicity (Diagne et al., 2013). The remaining 4% comprises marginal environments like mangroves and deep-water systems, where tidal fluctuations and pest infestations, notably rice yellow mottle virus (RYMV), severely limit productivity (Rodenburg et al., 2014). Despite over 230 million hectares of suitable land available for rice cultivation in SSA, only 12–14 million hectares are cultivated, with smallholder yields stagnating at <2 tons/ha, less than half the global average (OECD-FAO, 2021). Climate change exacerbates these challenges, with projections indicating 10-20% yield losses by 2050 due to heat stress, prolonged droughts, recurring floods, and invasive pests (AGRA, 2022). AfricaRice and its national partners have been implementing regional breeding programs for more than three decades, targeting the major rice-growing ecologies of sub-Saharan Africa. The breeding strategy emphasizes the development and dissemination of stress-tolerant, high-yielding, and farmer-preferred varieties for rainfed and irrigated systems through multi-environment testing across representative sites. Collaborative efforts under the Africa-wide Rice Breeding Task Force (AfricaRice-BTF) have contributed to the release of several improved varieties, including the NERICA and ARICA series, which address key production constraints such as drought, flooding, iron toxicity, and low soil fertility. Multi-environment trials (METs) are fundamental to any breeding programs. The expression of traits across environments can differ substantially by genotype, a phenomenon referred to as genotype by environment interaction (GEI) in METs. By modelling GEI, METs exploit inter-environment correlations to enhance the selection of stable, broadly adaptable genotypes, offering significant advantages over single-environment testing (Crossa, 2012; Hassen et al., 2018). Evaluating new genotypes across varied environments allows breeders to elucidate trait-environment interactions, facilitating the identification of cultivars with superior productivity and consistent performance across the target population of environments (TPE). Breeders employ a range of statistical approaches to identify and recommend cultivars suited for target environments. The breeding values of genotypes evaluated across multiple environments, estimated from mixed models, provide a robust basis for selection by accounting for genetic relationships and unbalanced trial data (Piepho et al., 2008; Bernardo, 2020). A commonly applied approach to exploit GEI is to regress genotype performance in each environment on the corresponding environmental mean, first introduced by Yates and Cochran (1938) and later established by Finlay and Wilkinson (1963). Several statistical frameworks have been employed to dissect GEI, including fixed linear models (e.g. factorial regression), bilinear models, linear–bilinear fixed‐effect models (e.g. AMMI; GGE/site regression), linear mixed models (e.g. multi-trait), and linear–bilinear mixed models (e.g. factor‐analytic models) (Crossa, 2012).

The estimation of genetic gain serves as a critical metric for evaluating the success and direction of plant breeding programs. It helps in assessing the progress in improving key traits such as yield, stress tolerance, and grain quality over time and informs the design of breeding strategies to accelerate future gains (Fufa et al., 2005). However, in SSA, varietal turnover remains sluggish; older, low-yielding varieties continue to dominate farmers’ fields. This slow uptake of improved varieties, despite their superior agronomic and grain quality traits, can be attributed to weak seed dissemination systems, limited farmer awareness, and gaps in agricultural policy and investment (Futakuchi et al., 2021).

To assess long-term genetic progress, the present study employed ERA trials conducted across multiple locations in three to five major rice-producing countries per network. ERA trials, pioneered by Duvick in studies published between 1972 and 2004, are now a well-established method for assessing genetic gain in cereal crops, especially where long-term breeding program data exists (Duvick, 2005; Rutkoski, 2019). Studies in crops like maize and wheat have applied this method effectively to estimate the genetic contribution to yield improvement by regressing variety performance against their year of release (Brancourt-Hulmel et al., 2003; Zheng et al., 2011; Sharma et al., 2012; Laidig et al., 2014). Similar approaches have been used to quantify progress in grain yield over time in rice in Asia (Peng et al., 2000) and in maize (Asea et al., 2023; Tarekegne et al., 2023) and wheat (Lopes et al., 2012) in Africa. To the best of our knowledge, this is the first study focused on estimation of genetic gain in grain yield of rice using ERA trials in SSA. While measuring gains using ERA trials has some limitations, firstly, released varieties may not fully represent the entire breeding populations, and the year of release of a variety does not represent the year of its creation. Nevertheless, they offer a practical and accessible means for estimating long-term genetic trends, especially in the absence of detailed annual breeding cycle data (Rutkoski, 2019). On the other hand, the advantage of using ERA trials based on released varieties to estimate genetic gains over a period is the fact that these trials are done on the final outputs of breeding programs (i.e., the varieties). The genetic gains estimation based on historical trials conducted over years by breeding programs has a limitation that the large number of those lines would never be released/cultivated. Therefore, despite their constraints, ERA trials are highly useful for estimating realized genetic gains in a way that aligns with varietal adoption and farmer impact. The realized gain, i.e., estimated based on released varieties/farmers’ fields, is more meaningful to farmers than gains estimated from experimental breeding lines (Atlin et al., 2017). While the ERA approach allows for estimation of realized genetic trends under uniform management conditions, it is important to note that testing older varieties in modern environments may introduce environmental bias. The yield performance of historical varieties may not fully represent their original on-farm performance due to advances in agronomic management, environmental shifts, and evolving pest and disease pressures. Nevertheless, ERA trials remain a practical framework for quantifying realized genetic progress in the absence of long-term, standardized breeding-cycle data sets (Duvick, 2005; Rutkoski, 2019).

This study aims to quantify the genetic trends in grain yield of rice varieties developed or released by AfricaRice and partners and grown in SSA, using performance data from ERA trials; and select the best performing and most stable genotypes. By analyzing yield trends over the past three decades, this study highlights the historical impact of AfricaRice’s breeding efforts and pinpoints areas where further genetic improvement is needed. These insights are essential for modernizing breeding programs and shaping their strategic direction, especially under the challenges of climate change, rapid population growth, shifting agricultural landscapes, and the need to support fragile economies in SSA.

## 2 Materials and methods

### 2.1 Breeding materials and experimental details

This study focused on rice varieties bred by the Africa Rice Center (AfricaRice) and released by partner countries between 1986 and 2020 across SSA as part of ERA trials. The trials were conducted under three distinct rice-growing ecologies: irrigated lowland, rainfed lowland and rainfed upland. A total of 36 released varieties were evaluated under the irrigated lowland ecology, 24 varieties under the rainfed lowland ecology, and 20 varieties under the rainfed upland ecology. Supplemental file_S1 highlights the list of varieties used in these trials along with their corresponding year of release. The trials were carried out over two wet seasons (2021 and 2022). All trials were established using an alpha lattice design with three replications to control for spatial variability and improve statistical accuracy.

For rainfed upland ecology, the trial was conducted in four locations: M’Bé (Côte d’Ivoire), Longolora and Mopti (Mali), and Ibadan (Nigeria), (**Table 1**). All tested varieties were planted using direct seeding methods in plots comprising 6 rows, with 4 meters length and 25 x 25 cm spacing between rows and plants (total plot area = 6 m²). Fertilizer was applied at rates of 100 kg/ha of nitrogen (N), 40 kg/ha of phosphorus (P_2_O_5_), and 40 kg/ha of potassium (K_2_O). For irrigated lowland ecology, trials were conducted in five locations in five countries: Vallée du Kou (Burkina Faso), M’Bé (Cote d’Ivoire), Kilissi (Guinea Conakry), Niono (Mali), and Ndiaye (Senegal). Regarding rainfed lowland ecology, the trials were conducted at five locations in five countries: Sérédou (Guinea Conakry), M’Bé (Cote d’Ivoire), Sikasso (Mali), Ibadan (Nigeria), and Fanaye (Senegal) (**Table 1**). For the last two ecologies, 21-day-old seedlings were transplanted in 6 rows with 4 m length, and spacing between rows and plants was maintained at 20 cm × 20 cm. NPK was applied at the rate of 120:60:40 kg ha^-1^ with N applied in three doses, first as basal, second at the tillering stage, and third at panicle initiation, while P and K were applied as basal in a single dose. Data collection included key agronomic and phenological traits such as days to flowering, days to maturity, plant height, and plot yield were measured (IRRI, SES, 2016). Plot yield was converted to tons/ha based on harvested hills or area, adjusted for the grain moisture content. While our study relied on only two experimental years, the robust multilocation design provided broad environmental representation across three major rice-growing ecologies, ensuring meaningful genotype-by-environment interactions despite limited temporal replication over multiple years.

**Table 1:** Description of field sites of ERA trials 2021-2022.

Ecology	Sites (Country)	Latitude	Longitude	T (°C)	RH (%)	P (mm)	Soil types
Irrigated lowland	Vallée du Kou (Burkina Faso)	11°24’36”N	-4°22’9.84”W	26	85	503	Hydromorphic
	M’Bé (Côte d’Ivoire)	7°57’39.89”N	-5°06’36.63”E	26	81	950	Clay
	Kilissi (Guinea Conakry)	9°55’59.99”N	-12°49’59.99”E	22	83	2800	Ferralitic & hydromorphic
	Niono (Mali)	14°16’55.17”N	-5°57’13.96”E	25	78	1243	Clay
	Ndiaye (Senegal)	16°12’3.6”N	-16°15’52.99”E	24	40	438	Clay
Rainfed lowland	Sérédou (Guinea Conakry)	8°53’16”N	10°26’30”W	24	81	3300	Ferralitic forest soil
	M’Bé (Côte d’Ivoire)	7°57’39.89”N	-5°06’36.63”E	26	81	950	Clay
	Sikasso (Mali)	11°21’53.42”N	-5°39’59.99”E	26	84	461	Clay loam
	Ibadan (Nigeria)	7°25′56”N	4°00′07”E	27	83	2566	Clay
	Fanaye (Senegal)	16°31’5.66”N	-15°11’56.86”E	22	50	738	Clay
Rainfed upland	M’Bé (Côte d’Ivoire)	7°57’39.89”N	-5°06’36.63”E	26	81	950	Clay
	Longolora (Mali)	11°21’59.10”N	-5°40’35.50”E	26	84	461	Sandy loam
	Mopti (Mali)	14°30’43.51”N	-4°14’3.20”E	25	83	1772	Sandy loam
	Ibadan (Nigeria)	6°50’58.8”N	3°42’20.6”E	27	83	2088	Clay loam

Weather data (T, Temperature in °C; RH, Relative Humidity in %; P, Annual precipitation in mm). Source: NASA POWER.

### 2.2 Statistical modelling

#### 2.2.1 Relationship among released varieties

Genetic similarity between the released varieties was assessed using the pedigree-based relationship matrix. Varietal diversity and similarity were visualized using a heatmap graph on the A-matrix also known as the Numerator Relationship Matrix (NRM) of released varieties. The NRM was performed using the R package *sommer* (Covarrubias-Pazaran, 2016), and the heatmap graph was drawn using the R package *heatmaply* (Galili et al., 2018).

#### 2.2.2 Estimation of the genetic trends

Breeding values were estimated by considering combinations of sites and years as a single trial or environment. The ERA 2021–2022 datasets were subjected to preprocessing and quality checks (filtering trials with unexpected phenotypic values and tagging extreme data points and outliers) to ensure that high-quality trials (H^2^ > 0.1 and r^2^ > 0.15) and phenotypes were retained for downstream analysis and estimation of breeding values and genetic gains. After pre-processing and quality control, the dataset comprised a total of 18 trials with 36, 24, and 20 unique varieties for irrigated lowland, rainfed lowland, and rainfed upland, respectively.

The two-step approach (Mackay et al., 2011; Laidig et al., 2014) was used to estimate the yield genetic trend of released varieties for each ecology. In the first step, the Best Linear Unbiased Estimates (BLUEs) of grain yield regarding individual (site × year combination) trials were extracted from a linear mixed model (LMM). In the LMM, varieties were considered as fixed effects, while replications and blocks, were considered as random effects. Trial heritability was estimated from LMM but by specifying varieties as random effects. The basic model used in the first step is given below:


$$y_{ijk}=\mu +r_{i}+b_{j}(r_{i})+g_{k}+Cov+\theta_{ijk}$$

where *y_ijk_* is the response variable grain yield, *μ* is the mean effect, *r_i_* is the effect of the *i*th replicate, *b_j_*(*r_i_*) is the effect of the *j*th incomplete block within the *i*th replicate, *g_k_* is the effect of the *k*th genotype (here variety), *Cov* is the effect of the covariate (days to flowering and plant height., and *θ_ijk_* is the error associated with the *i*th replication, *j*th incomplete blocks, and the *k*th genotype, which is assumed to be normally and independently distributed, with mean zero and homoscedastic variance *σ*^2^*^θ^*.

The broad-sense heritability (H^2^) was estimated using (Cullis et al. (2006) and (Piepho and Möhring, 2007) methods, as below:


$$H_{Cullis}^{2}=1-\frac{{\bar{v}}_{BLUP}}{2\sigma_{g}^{2}}\;;\;H_{Piepho}^{2}=\frac{\sigma_{g}^{2}}{\sigma_{g}^{2}+\frac{{\bar{v}}_{BLUE}}{2}}$$

Where *σ^2^* refers to variance, *g* to genotype, ${\bar{\upsilon }}_{BLUP}$ to the average variance of a difference of two genotypic BLUPs, and ${\bar{\upsilon }}_{BLUE}$ to the average variance of a difference of two genotypic BLUEs.

In the second step, for a given ecology, the BLUEs from the first step were used as a response variable. In this step, the previous A matrix was fitted for the rainfed upland to account for the genetic covariances among the varieties for reliable estimates of breeding values. The same model was used for the irrigated lowland and rainfed lowland without fitting the A matrix to extract the new BLUEs. Indeed, for irrigated and rainfed lowland, the models raised singularity issues and failed. This often suggests that some aspects of the random effects are perfectly correlated or have zero variance, indicating that they don’t add new information or explain any additional variation in the data. The model fitted in the second step is as follows:


$$y_{ij}=\mu +g_{i}+y_{j}+g_{i}\times y_{j}+\theta_{ij}$$

Where *y_ij_* is the adjusted mean weighted by the standard errors for the *i*th genotype in the *j*th trial, *μ* is the overall mean, *g_i_* is the breeding value of the *i*th genotype with *g_i_*~*N*(*ζ*, *A_σ_^2^g*) where *σ^2^g* is the genetic variance and A is the NRM, *y_j_* is the fixed effect of the *j*th trial, *g_i_* × *y_j_* is the random effect of genotype by environment interaction, and *θ_ij_* is the residual error, with *θ_ij_*~*N*(0, *Rσ^2θ^*), where *R* is the identity error covariance matrix and *σ*^2^*θ* is the error variance.

To be realistic, the variance-covariance matrix of the random genetic by environment effect is parametrized using the second-order factor analytic (FA2) model. According to Piepho (1998), the FA2 model is known to adjust genetic heteroscedasticity in a parsimonious manner. The first and second step models were fitted using the R package asreml (The VSNi Team, 2023). The factor-analytic model was used to describe how varieties performed differently across locations and years while keeping the analysis straightforward. This approach helps to capture the main patterns of genotype-by-environment interaction without making the model too complex. We carefully checked the model outputs and ensured that the results were stable and reliable.

Moreover, the genotype by environment (G × E) tables of the estimated means resulting from the first step model were used to calculate the Finlay–Wilkinson model (Finlay and Wilkinson, 1963) with a single regression line on the environmental quality in the model μ*_ij_* = μ + *G_i_ + E_j_ + b_i_E_j_* + ϵ*_ij_*. From the model, *b_i_* represents the environmental adaptability or sensitivity (Malosetti et al., 2013). The Finlay–Wilkinson regression was used as a simple measure of yield stability and varietal responsiveness to environmental quality, complementing the mixed-model results. It was used to interpret varietal adaptation patterns across environments. Given the limited dataset, these models were applied primarily for descriptive purposes, and results were interpreted cautiously.

The regression terms for the time trends for the main effects of the genotype effect *g_i_* and the year effect *y_k_* are then incorporated into the model. The main effect of genotype *g_i_* is modelled as a function of the year of release (*a_i_*) as follows:


$$g_{i}=\gamma +\beta a_{i}+\tau_{i}$$

where, γ is the regression intercept (the grand mean across all genotypes), *β* is the regression slope for the genetic trend, and *τ_i_* is the random deviation from the genetic trend line with *τi ~ N(*0*, σ^2^τ)*. This regression model was fitted using the R package stats (R Core Team, 2024).

The percentage change in genetic gain, %*gg*, can be divided into the first-year part, %*gg*(*a*_1_), and the average-year part, %*gg*(*ā*). The %*gg* per year for each level of ecology associated with the genetic causes was estimated as follows (Kumar et al., 2021):


$$\%gg(a_{1})=\frac{\beta }{\gamma +(\beta \times a_{1})}\times 100$$

$$\%gg(\bar{a})=\frac{\beta }{\gamma +[\beta \times \frac{\left(a_{1}+a_{n}\right)}{2}]}\times 100$$

where, γ and *β* are defined as previously, *a*_1_ and *a*_n_ are, respectively, the beginning and end of the period over which the genetic trend was evaluated. Plots of genetic trends were drawn using the R packages ggplot2 (Wickham, 2016) and ggpmisc (Aphalo, 2023). All statistical analyses were performed using R software version 4.3.3 (R Core Team, 2024).

## 3 Results

### 3.1 Diversity/similarity among released varieties

**Figure 1:**
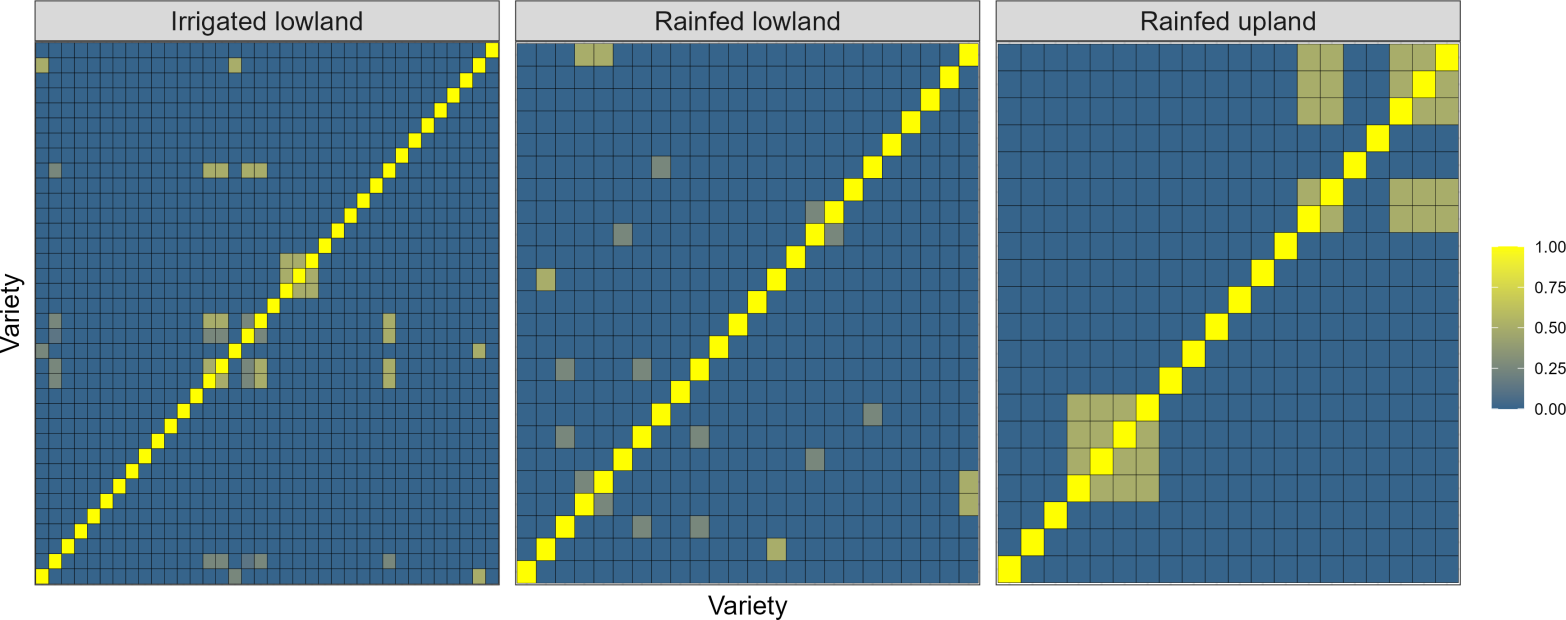
Heatmap of Numerator Relationship Matrix (NRM) of released varieties.

The kinship matrices of ERA varieties were plotted in heatmap format for each breeding ecology (**Figure 1**). Irrespective of the ecology, there was no relationship between varieties from different families. The heatmap highlighted ~32, 22, and 14 sibships for irrigated lowland, rainfed lowland, and rainfed upland, respectively. In the irrigated and rainfed lowland ecologies, most varieties were only weakly related, with a few small clusters, suggesting that selection has drawn from a broad genetic base. In contrast, rainfed upland varieties formed two clear clusters, indicating a narrower genetic base and heavier reliance on a limited number of diverse lines.

### 3.2 Genetic variabilities and mean performances

**Figure 2:**
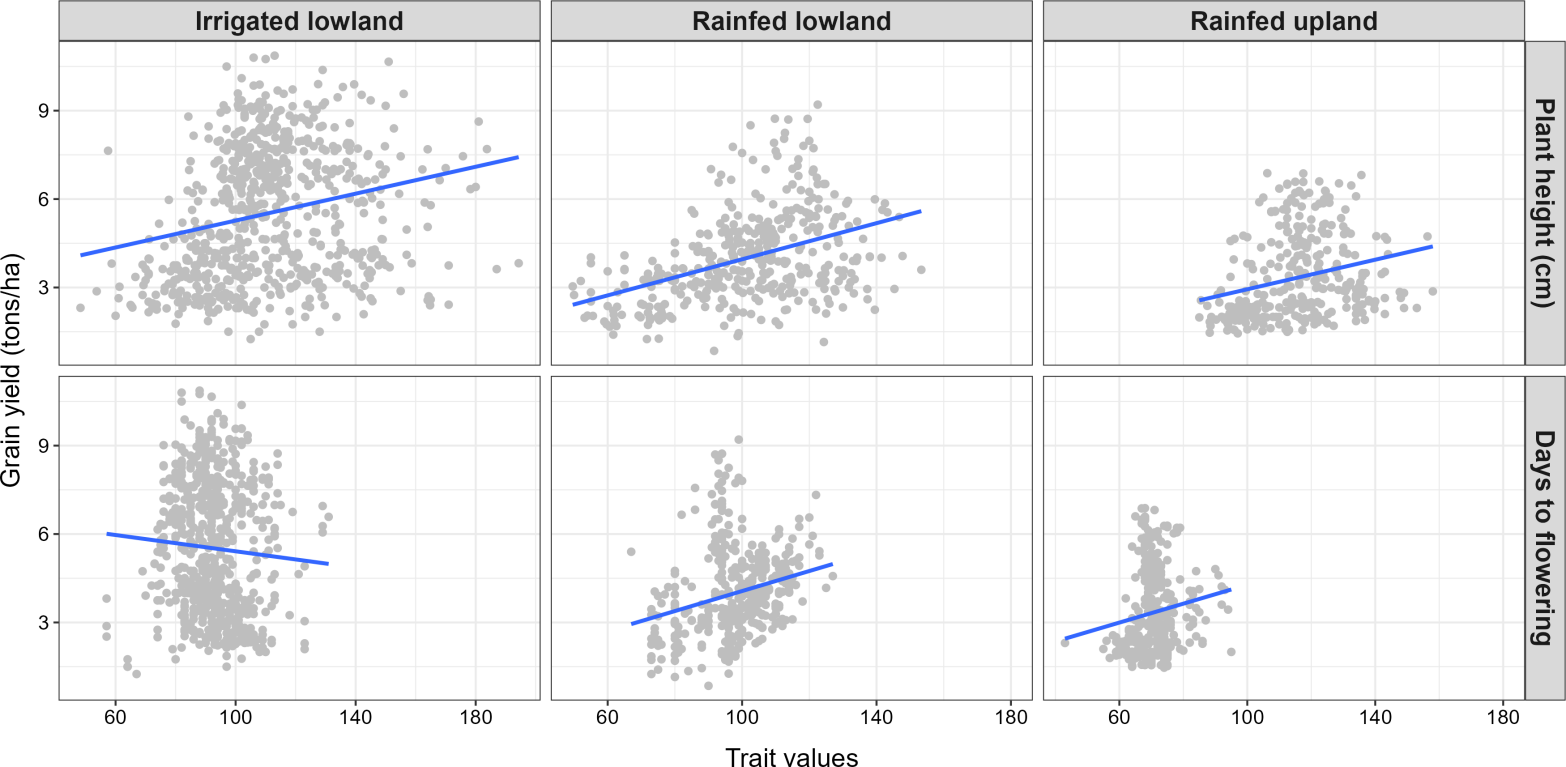
Evolutionary trends in grain yield with covariates (days to flowering and plant height).

The relationship between covariates and grain yield is summarized in **Figure 2**. Irrespective of ecology, plant height was positively correlated with grain yield (correlation coefficient of 0.23, 0.26 and 0.42 respectively in irrigated lowland, rainfed lowland and rainfed upland). Regarding days to flowering, a positive correlation was observed with grain yield under rainfed ecologies (correlation coefficient of 0.15 and 0.24 respectively for upland and lowland). Under irrigated lowland, increasing days to flowering resulted in decreasing grain yield (correlation coefficient of -0.06).

The average grain yield by trial ranged from 2.766 tons/ha in Kilissi (Guinea Conakry) to 8.271 tons/ha in Niono (Mali) under the irrigated lowland ecology, 2.353 tons/ha in Seredou (Guinea Conakry) to 6.298 tons/ha in Sikasso (Mali) under rainfed lowland ecology, and 2.156 tons/ha in Longolora (Mali) to 5.552 tons/ha in Mopti (Mali) under rainfed upland ecology. The trials with broad sense heritability (H^2^) greater than 0.1 were retained for subsequent analysis, according to Johnson et al. (1955). The H^2^ of the irrigated lowland, rainfed lowland, and rainfed upland trials retained for the analysis ranged from 0.300 to 0.835, 0.308 to 0.936 and 0.244 to 0.728, respectively (**Table 2**).

**Table 2:** Performance (trial yield mean, broad sense heritability and reliability: R^2^) of release varieties from single trial analysis across ERA trials 2021-2022.

Ecology	N°	Year	Location	Grain yield (tons/ha)	Heritability		R2
					*Cullis	^Piepho	
Irrigated lowland	1	2021	Kilissi (Guinea Conakry)	2.766	0.308	0.300	0.237
	2	2021	M’Be (Cote d’Ivoire)	3.598	0.481	0.436	0.465
	3	2021	Ndiaye (Senegal)	6.526	0.731	0.552	0.730
	4	2021	Niono (Mali)	8.271	0.751	0.751	0.749
	5	2021	Vallee du Kou (Burkina Faso)	3.571	0.835	0.793	0.835
	6	2022	Kilissi (Guinea Conakry)	3.311	0.439	0.421	0.413
	7	2022	M’Be (Cote d’Ivoire)	7.528	0.701	0.652	0.699
	8	2022	Niono (Mali)	6.038	0.466	0.468	0.448
Rainfed lowland	1	2021	Fanaye (Senegal)	2.753	0.936	0.930	0.937
	2	2021	Seredou (Guinea Conakry)	2.353	0.407	0.398	0.357
	3	2021	Sikasso (Mali)	6.298	0.648	0.575	0.642
	4	2022	Ibadan (Nigeria)	4.893	0.487	0.364	0.462
	5	2022	Seredou (Guinea Conakry)	3.624	0.308	0.320	0.219
	6	2022	Sikasso (Mali)	4.102	0.619	0.617	0.611
Rainfed upland	1	2021	Longolora (Mali)	2.156	0.615	0.485	0.614
	2	2021	M’Be (Cote d’Ivoire)	2.640	0.521	0.475	0.503
	3	2022	Ibadan (Nigeria)	3.494	0.728	0.726	0.724
	4	2022	Mopti (Mali)	5.552	0.330	0.244	0.270

*Cullis et al. (2006), ^Piepho and Möhring (2007).

**Figure 3:**
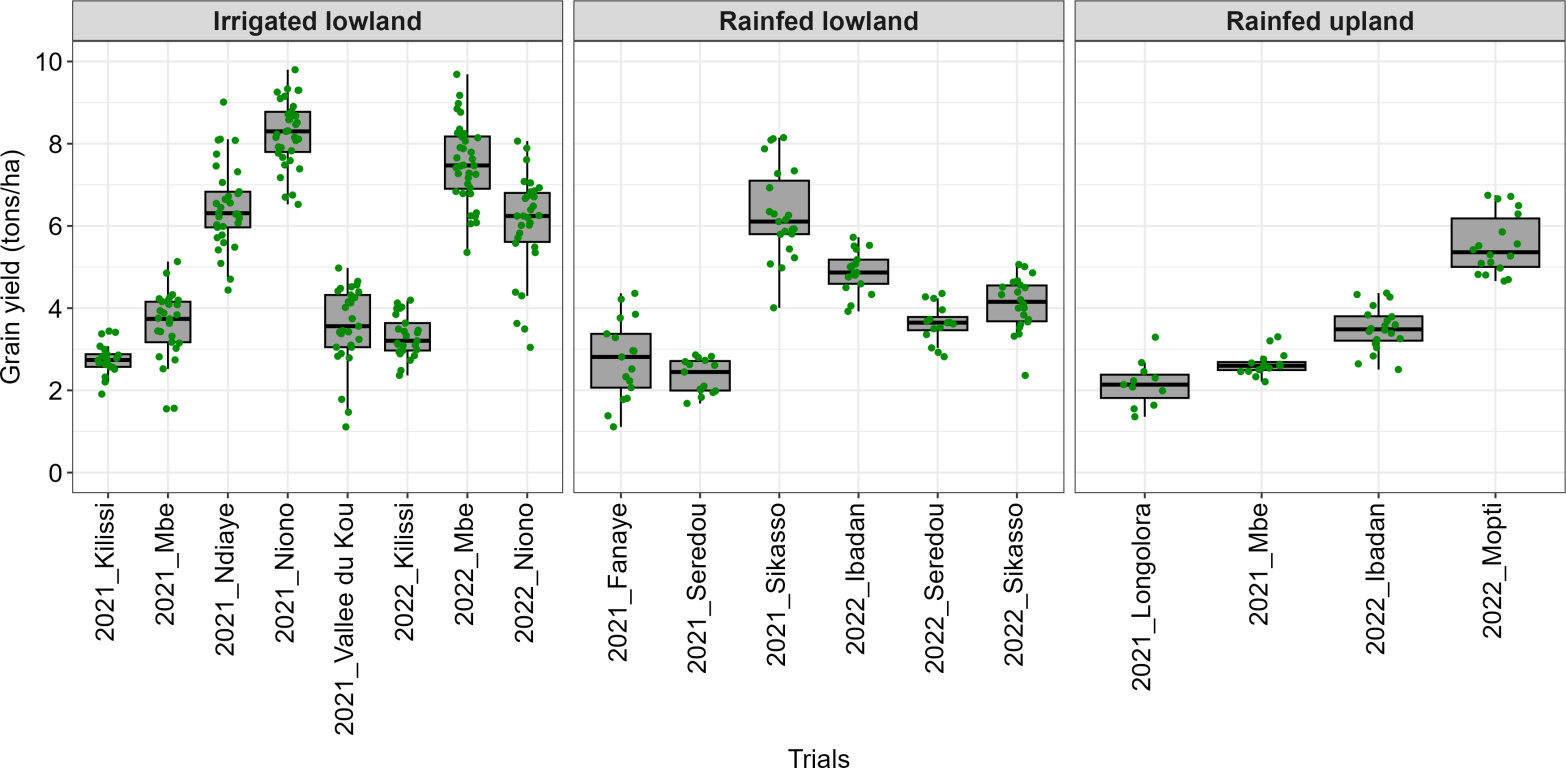
Boxplots of Best Linear Unbiased Estimates (BLUEs) for grain yield from single-trial analyses across ERA trials, 2021–2022.

The boxplots of the Best Linear Unbiased Estimates (BLUES) derived from the grain yield (tons/ha) showed notable variations among the tested varieties across the various locations under each ecology (**Figure 3**). The widest variability (coefficient of variation of 27%) under the irrigated lowland ecology was observed at Vallee du Kou (Burkina Faso), while the lowest variability (coefficient of variation of 10%) was obtained at Niono (Mali) in 2021. Under the rainfed lowland ecology, the largest variability (coefficient of variation of 35%) was obtained at Fanaye (Senegal), while the lowest variability (coefficient of variation of 10%) was obtained at Ibadan (Nigeria) in 2022. Regarding the rainfed upland ecology, the largest variability (coefficient of variation of 25%) was obtained at Longolora (Mali), while the lowest variability (coefficient of variation of 11%) was obtained at M’Bé (Côte d’Ivoire).

### 3.3 Genetic correlations and genetic trend estimates

**Figure 4:**
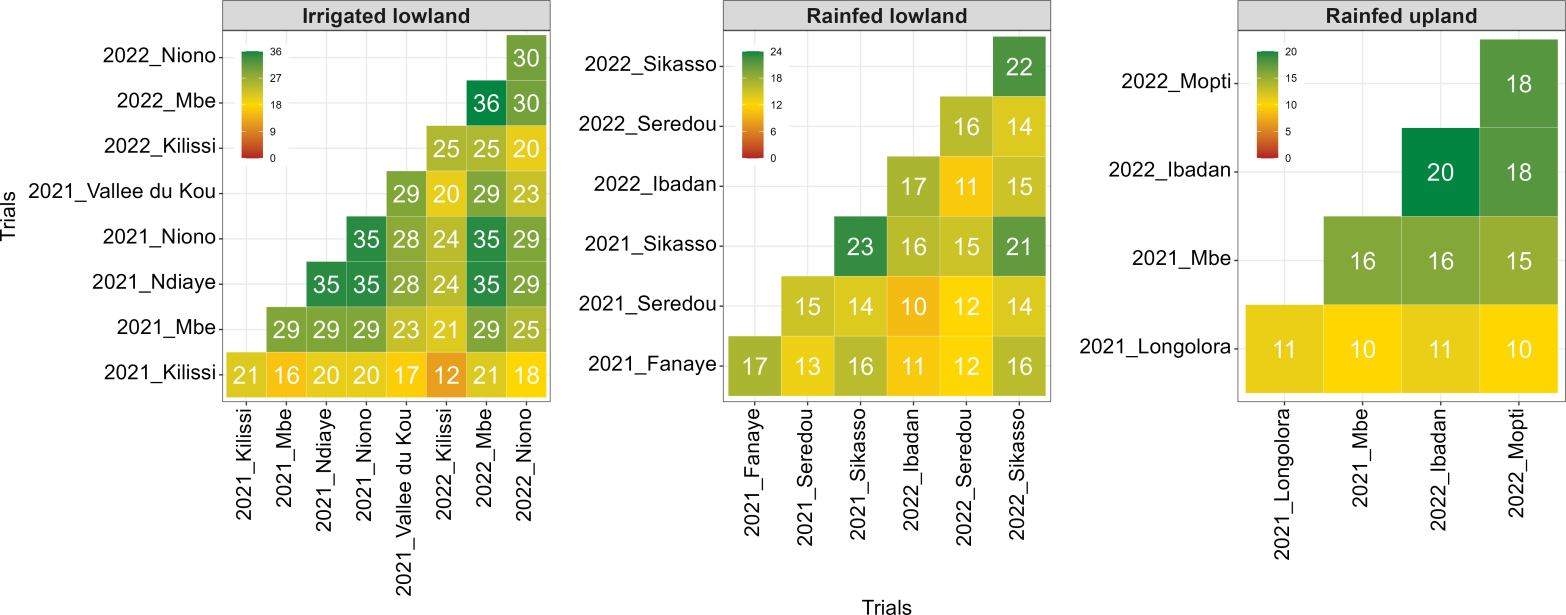
Genotype connectivity across environments from single trial analysis across ERA trials, 2021-2022.

The connectivity between environments is summarized in **Figure 4**. It ranged from 10 genotypes for Longora (Mali) × Mbe (Côte d’Ivoire) to 18 genotypes for Ibadan (Nigeria) × Mopti (Mali) under rainfed upland and from 10 genotypes for Seredou (Guinea Conakry, 2021) × Ibadan (Nigeria) to 21 genotypes for Sikasso (Mali, 2021 × 2022) under rainfed lowland. With regards to irrigated lowland, the connectivity ranged from 12 genotypes for Kilissi (Guinea Conakry, 2021 × 2022) to 35 genotypes for N’Diaye (Senegal) × Niono (Mali, 2021) and N’Diaye (Senegal) × M’Be (Côte d’Ivoire, 2022).

**Figure 5:**
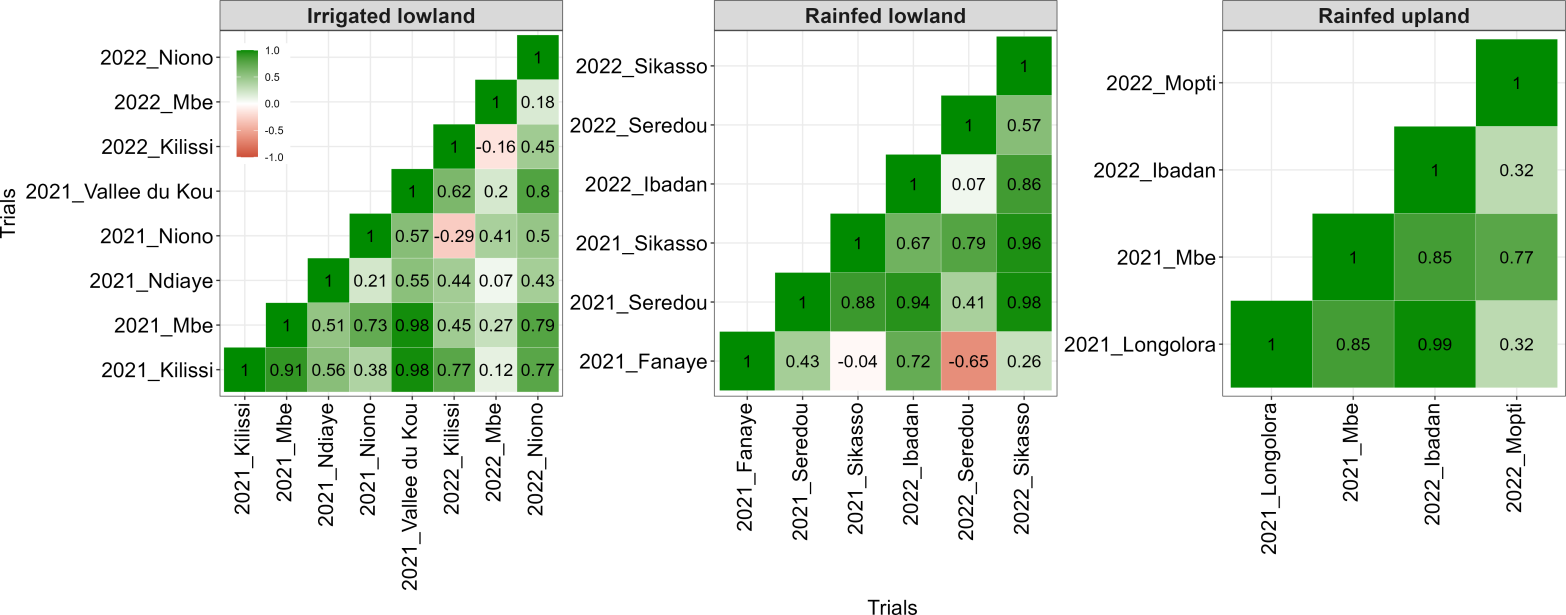
Genotypic correlation between trials/environments using second-order factor analytic model.

Genetic correlations (*r*) for grain yield were positive between almost all pairs of environments irrespective of ecology (*r* = 0.07-0.98 for irrigated lowland, *r* = 0.07-0.98 for rainfed lowland, *r* = 0.32-0.99 for rainfed upland), indicating that environments can be combined to estimate breeding values to make relevant predictions in the modelling process (**Figure 5**). However, negative correlations (*r* = -0.29 and *r* = -0.16 respectively) were observed for Kilissi (Guinea Conakry, 2022) with Niono (Mali, 2021), and 2022-M’Be (Cote d’Ivoire, 2021) for irrigated lowland. A genetic correlation of -0.65 was observed between Fanaye (Senegal) and Seredou (Guinea Conakry, 2022) for rainfed lowland. The connectivity among test environments was relatively low because of differences in release years, ecology-specific adaptation, and seed availability, which limited the number of varieties shared across sites. As a result, the high genetic correlations observed for grain yield across environments should be interpreted with caution. These values may reflect restricted genotype overlaps and limited environmental contrast rather than true biological stability. Similar constraints are common in ERA datasets based on released varieties (Peng et al., 2000; Asea et al., 2023).

**Figure 6:**
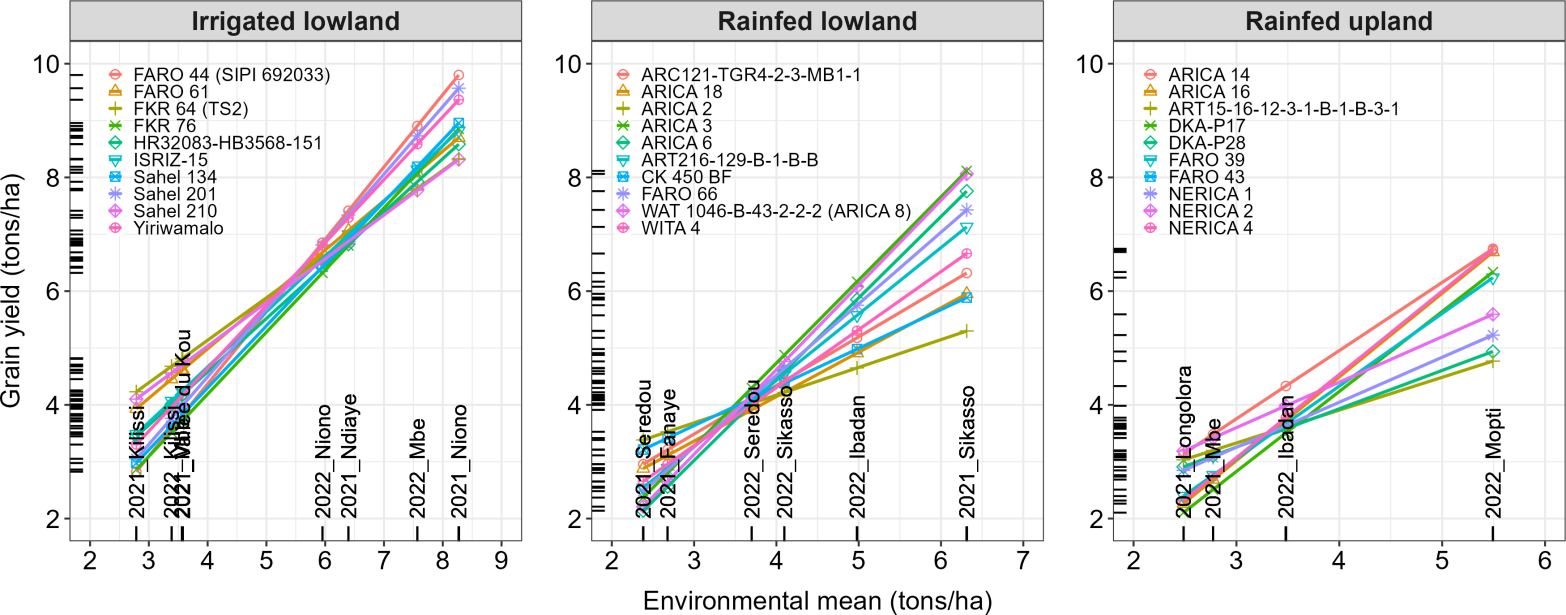
Finlay–Wilkinson genotype-by-environment plots for top-performing released varieties across irrigated lowland, rainfed lowland, and rainfed upland ecologies.

The Finlay–Wilkinson model showed a significant (*p-value* < 0.05) genotype by environment interactions for rainfed lowland and rainfed upland (**Table 3**). Indeed, in **Figure 6**, WITA 4, ARICA 18, ARC121-TGR4-2-3-MB1-1, CK 450 BF and ARICA 2 were the best performing varieties (grain yield ≈ 2.7-3.4 tons/ha) at Seredou (Guinea Conakry) in 2021 while ART216-129-B-1-B-B, FARO 66, ARICA 6, ARICA 8 and ARICA 3 were best (grain yield ≈ 7.1-8.1 tons/ha) at Sikasso (Mali) in 2021 under rainfed lowland. With regards to rainfed upland, varieties NERICA 1, DKA-P28, ART15-16-12-3-1-B-1-B-3-1, ARICA 14 and NERICA 2 registered the best performance (grain yield ≈ 2.8-3.2 tons/ha) at Longolora (Mali) in 2021 while FARO 39, DKA-P17, ARICA 16, NERICA 4 and ARICA 14 were best (grain yield ≈ 6.2-6.7 tons/ha) at Mopti (Mali) in 2022. Although the analysis of variance (**Table 3**) showed a non-significant (*P-value* > 0.05) genotype by environment interaction for irrigated lowland, the Finlay–Wilkinson model plot revealed an existing interaction. For instance, the best performing varieties included HR32083-HB3568-151, ISRIZ-15, FARO 61, Sahel 210 and FKR 64 (grain yield ≈ 3.4-4.2 tons/ha) in Kilissi (Guinea Conakry) in 2021, while FKR 76, Sahel 134, Yiriwamalo, Sahel 201 and FARO 44 were the best performing varieties (grain yield ≈ 8.9-9.8 tons/ha) in Niono (Mali) in 2021.

**Table 3:** Analysis of variance with subdivision of variety × environment interactions using regression analysis on grain yield variety × environment means following the Finlay–Wilkinson model.

Source of variation	Irrigated lowland			Rainfed lowland			Rainfed upland		
	Df	*F-value*	*P-value*	Df	*F-value*	*P-value*	Df	*F-value*	*P-value*
Environment	7	225.018	< 0.001	5	102.297	< 0.001	3	238.856	< 0.001
Variety	35	4.895	< 0.001	23	3.138	< 0.001	19	9.765	< 0.001
Regression	35	0.872	0.675	23	2.023	0.016	19	2.110	0.045
Error	160			58			23		

Df, Degree of freedom.

**Figure 7:**
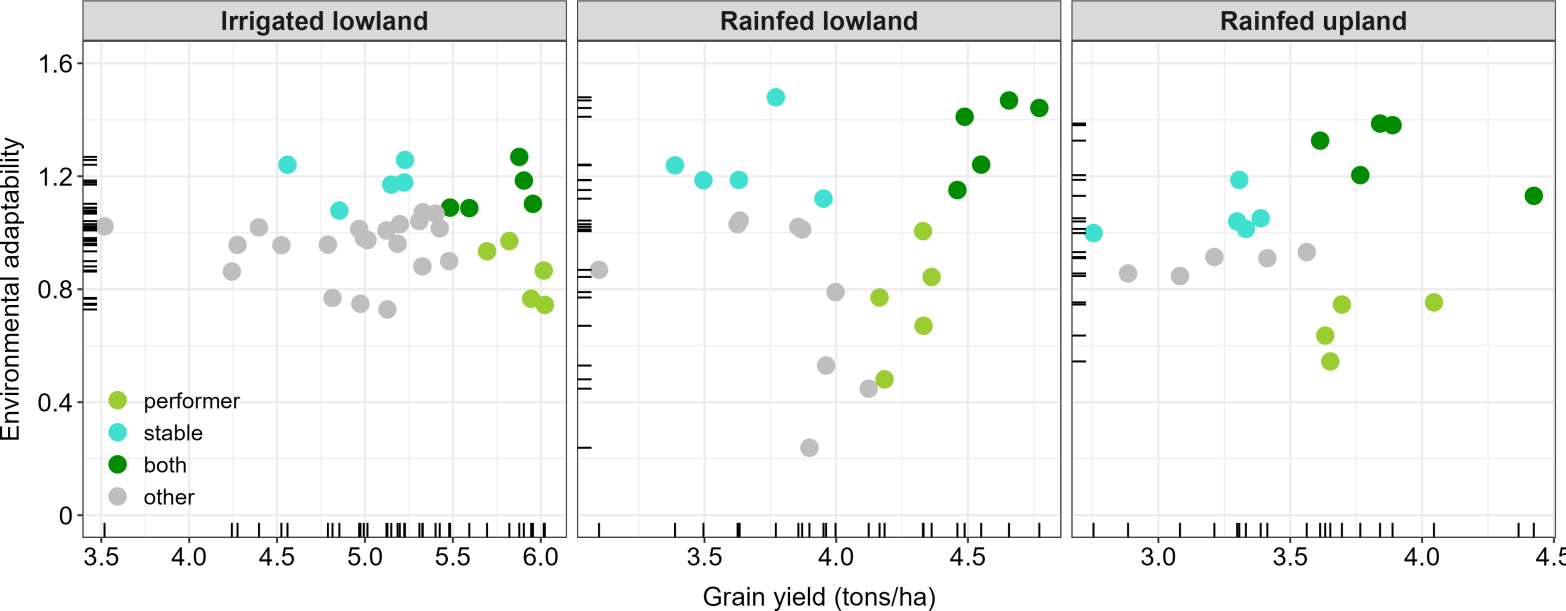
Finlay–Wilkinson model adaptability or stability *versus* performance plots for irrigated lowland, rainfed lowland and rainfed upland released varieties.

Moreover, the Finlay–Wilkinson model adaptability or stability *versus* performance plot (**Figure 7**) revealed that Yiriwamalo, Sahel 101, FARO 44, Sahel 134 and FKR 76 were the most yielding (grain yield ≈ 5.5-6.0 tons/ha) and stable (stability index ≈ 1.1-1.2) varieties across environments under irrigated lowland while ARICA 3, ARICA 8, FARO 66, ARICA 6 and ART216-129-B-1-B-B were the most yielding (grain yield ≈ 4.5-4.8 tons/ha) and stable (stability index ≈ 1.2-1.5) varieties across environments under rainfed lowland. Regarding rainfed upland, the most yielding and stable varieties included ARICA 14, NERICA 4, ARICA 16, FARO 39 and DKA-P17 (grain yield ≈ 3.6-4.4 tons/ha and stability index ≈ 1.1-1.4).

**Figure 8:**
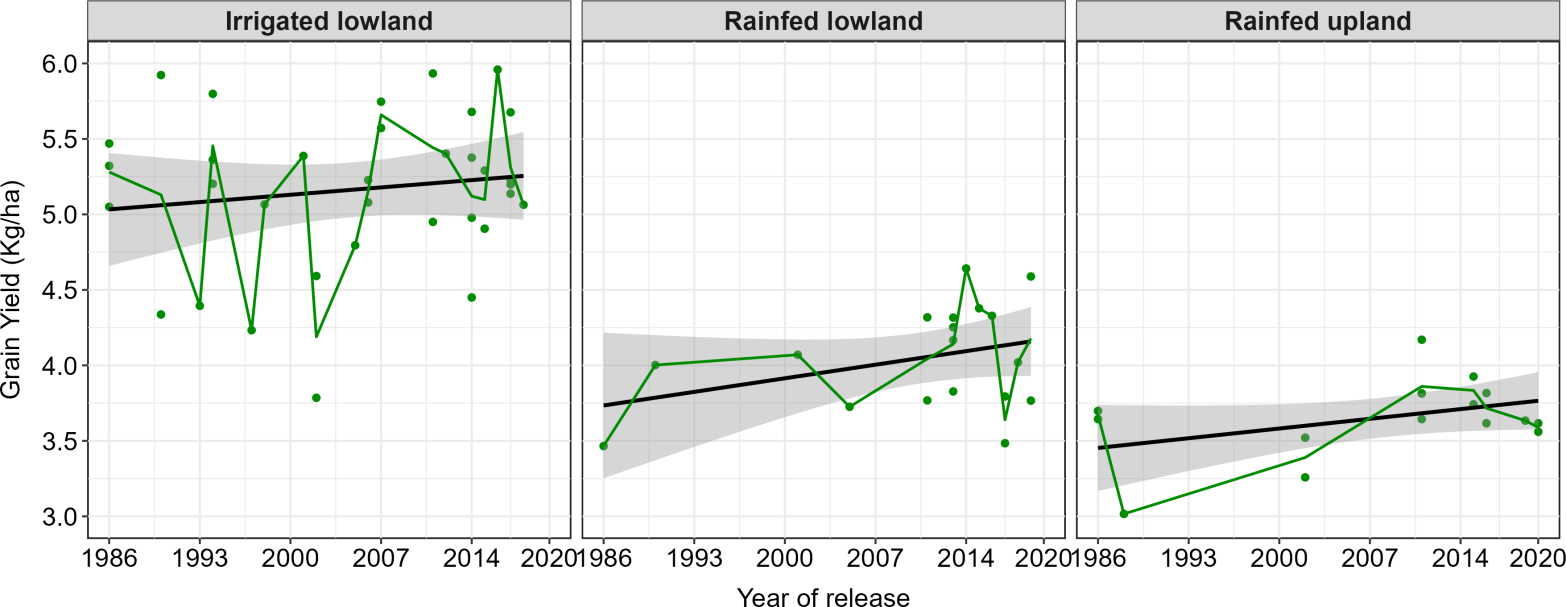
Genetic trends in grain yield of released varieties in Africa. The dots represent the variety performance; the green lines represent their group means from the analysis across varieties, and the black lines represent regression lines.

**Table 4** and **Figure 8** summarize the genetic trends in released rice yields across ecologies. In irrigated lowland, the trend was positive, with a yield gain of 7 kg/ha/year, corresponding to 0.14% annual genetic gain. In rainfed lowland, the trend was 12 kg/ha/year (0.34% per annum). Similarly, in rainfed upland, a positive trend of 10 kg/ha/year was observed, representing 0.27% annual genetic gain. The variability in genotype mean performance across ecologies was reflected in the fluctuations of group means. Large fluctuations were observed under irrigated lowland conditions, indicating greater variability among genotypes. In contrast, relatively lower fluctuations were observed in rainfed lowland and upland ecologies, suggesting more uniform genotype performance in these environments. Confidence intervals showed wide ranges 4 to14, 1 to 23, and 2 to18 kg ha^−1^ yr^−1^ of gain under irrigated lowland, rainfed lowland and rainfed upland respectively. Despite these broad ranges, the positive slope across all ecologies suggests continuous, though gradual, genetic progress in yield over the past three decades.

**Table 4:** Estimates of regression coefficients for the genetic trends.

Parameters	Irrigated lowland		Rainfed lowland		Rainfed upland	
	Estimate	Std.err	Estimate	Std.err	Estimate	Std.err
Regression coefficient						
γ	-8.729	17.070	-21.787	17.415	-14.790	10.726
β	0.007	0.009	0.013	0.009	0.009	0.005
*R* ^2^	0.020	–	0.121	–	0.115	–
Genetic gain						
$\%gg(s_{1})$	0.138 (0.007-0.269)	0.169	0.344 (0.034-0.654)	0.232	0.266 (0.058-0.474)	0.155
$\%gg(\bar{s})$	0.135 (0.005-0.261)	0.166	0.326 (0.032-0.619)	0.219	0.255 (0.055-0.454)	0.148

γ, regression intercept; β, regression slope; $\%gg(s_{1}),$ first year percentage change in genetic gain; $\%gg(\bar{s}),$ average percentage change in genetic gain.

## 4 Discussion

This study evaluated the genetic trends in AfricaRice’s rice breeding programs across irrigated lowland, rainfed lowland, and rainfed upland ecologies, using 2 years of multilocation data of AfricaRice and partners-bred/released varieties between 1986 and 2020 in Africa through “ERA” trials. Indeed, ERA trials are known as one of the accurate methods to estimate genetic gain (Rutkoski, 2019). In this study, there was full connectivity between the trials. A common challenge in using historical data for estimating genetic gain is the lack of connectivity among varieties and across trials, which limits the precision of genetic trend estimates (Seck et al., 2023). In this study, we observed minimal genetic relatedness among varieties across ecologies. To mitigate this, we incorporated when relevant, pedigree-based relationships matrix in the second stage of modelling to improve covariance structure and enhance the connectivity among varieties, thereby improving the accuracy of breeding value estimates, an approach also supported by previous studies (Juma et al., 2021; Khanna et al., 2024).

The irrigated and rainfed lowland ecologies showed mostly low off‐diagonal relatedness, indicating a broad genetic base among released varieties. In contrast, varieties in the rainfed upland ecology formed more pronounced clusters, reflecting higher relatedness and a narrower genetic base signals a potential plateau in future gain unless additional germplasm from other sources/programs is incorporated and prioritize broadening the genetic base to increase response to selection. These findings have implications for both the estimation of realized genetic gain and the potential for future improvements. Previous studies support these observations and highlights both opportunities and constraints in breeding programs under differing ecologies. For instance, population improvement via recurrent selection reported in upland rice program of Embrapa, Brazil showed high realized genetic gain (3.08%/yr) when recurrent selection was applied over cycles, but the gains were constrained until the genetic base was sufficiently broad (Pereira de Castro et al., 2023). Similarly, analyses of irrigated rice breeding program in Bangladesh revealed a positive baseline genetic gain for yield (~0.28%/yr) even where between-line variation was modest, highlighting that incremental progress is possible when selection is effective (Rahman et al., 2023). Studies in other crops also emphasize that incorporating pedigree or genomic relationships into multi-environment trial analyses improves the partitioning of additive variance and the accuracy of realized gain estimates (Velazco et al., 2019).

Assessing genetic gain remains a critical tool to evaluate breeding program efficiency, selection intensity, and prediction accuracy and serves as a key performance indicator for long-term improvement (Fufa et al., 2005). Sustained genetic gain, along with the preservation of genetic diversity, is central to the success of breeding programs (Cowling et al., 2017; Lehermeier et al., 2017; Gorjanc et al., 2018; Allier et al., 2019 and 2020). The interpretation of realized genetic gain is often confounded by G×E interactions, particularly under heterogeneous conditions like in SSA. In rainfed systems, fluctuations in water availability, soil fertility, and temperature stress frequently alter genotype rankings, masking or exaggerating yield progress depending on the trial environment (Atlin et al., 2017). Even in more stable irrigated systems, G×E remains relevant, as interactions with management practices or seasonal variability can distort estimates of genetic gain. Consistent with these observations, the current study found significant G×E interactions under rainfed conditions for both upland and lowland ecologies, while G×E interactions were non-significant under irrigated conditions. The positive yield trends are promising, however interpreted cautiously, as realized genetic gain may reflect a combination of genetic improvement and local environmental adaptation. This emphasizes the need for multi-environment testing in well-defined TPE and advanced statistical methods, such as factor-analytic mixed models (Piepho, 1998; Cooper et al., 1999), to explore G×E effects and provide accurate estimates of genetic gain, effectively distinguishing true genetic progress from environmental noises. It should be recognized that the relatively small number of varieties and environments in present study limits the precision of the G×E interaction estimates. The models were therefore used to explore general adaptation trends rather than to draw definitive statistical inferences. Despite these limitations, the consistent patterns observed across analytical approaches provide useful preliminary insights into varietal performance stability as previously reported by Peng et al. (2000) and Asea et al. (2023).

It is important to note that improvements in agronomic practices, mechanization, and input use over time can influence yield levels. However, in ERA trials, all varieties are evaluated under the same management conditions within each environment, which helps to minimize these non-genetic effects. Therefore, the yield differences observed among varieties mainly reflect genetic improvement rather than differences in agronomic management (Duvick, 2005; Atlin et al., 2017). The present study focused on estimating realized genetic gain using ERA trials based on released varieties, which represent the final outputs of breeding programs and are directly relevant from a farmer’s perspective. The relatively small dataset used in this study (total 80 varieties evaluated released over 34 years span for 3 major rice ecologies) reflects the full set of AfricaRice-bred varieties for which quality seed and release information were available across the three major rice ecologies. Although the number of entries may appear limited, this collection provides a valuable continental baseline for monitoring realized genetic progress over time. Comparable sample sizes have been used in earlier ERA studies on rice and maize, where the focus was on representativeness and data consistency rather than volume of lines/varieties evaluated (Peng et al., 2000; Asea et al., 2023).

Grain yield in rice significantly influenced by plant height and days to maturity (Peng et al., 1999). Yield gains were associated with a marked reduction in plant stature, consistent with the introduction of semi-dwarf genes such as sd1, which reduced lodging risk and improved harvest index (Peng and Khush, 2003; Siddiq and Vemireddy, 2021). Similarly, shorter maturity duration often targeted with higher yields, reflecting breeding strategies for intensification and climate adaptation. Several previous studies in Asia reported that reduced growth duration supports multiple cropping while maintaining competitive yield levels (Fukai and Cooper, 1995; Peng et al., 2000). However, excessively early maturity may limit assimilate accumulation and yield potential under favorable environments, highlighting the need for careful optimization of phenology (Lafitte et al., 2004; Dingkuhn et al., 2015). Similarly, excessive reduction in stature can restrict biomass accumulation and limit yield potential as plant height is closely associated with light interception and assimilate production (Khush, 2001; Peng et al., 1999). Overall, the negative associations of yield with plant height and maturity time illustrate how selection for shorter, lodging-resistant, and earlier-maturing ideotypes has underpinned realized genetic gains in rice (Kumar et al., 2007; Tabien et al., 2008; Breseghello et al., 2011; da Costa et al., 2021; Juma et al., 2021). Although the annual yield gains observed in this study (7–12 kg ha^−1^ yr^−1^) appear modest, they are within the range reported in other genetic gain studies on rice conducted under challenging environments (Kumar et al., 2021; Rahman et al., 2023; Khanna et al., 2024). These modest but steady gains indicate continuous genetic improvement despite the production challenges in sub-Saharan Africa. The wider confidence intervals highlight uncertainty due to the limited dataset and heterogeneous test environments, yet the directionally stable trends suggest that cumulative breeding efforts delivered incremental genetic improvements. The relatively higher gain in rainfed lowland environments likely reflects targeted selection for tolerance to major abiotic stresses such as drought, flooding, and iron toxicity, which are central to AfricaRice’s breeding priorities. In contrast, the smaller gains in irrigated lowland may indicate a slower varietal turnover and a narrowing margin for improvement under managed conditions.

In the current study, plant height & days to flowering was studied as covariate with grain yield. Plant height showed a positive correlation with grain yield across various ecologies. Days to flowering exhibited a positive correlation with yield in rainfed upland and lowland ecologies but a slight negative correlation (r = -0.06) in irrigated systems. These findings highlight critical trade-offs in rice yield. These findings underscore the trade-offs in rice improvement: while semi-dwarfs, early maturing ideotypes have driven global yield gains, optimizing height and maturity to match target environments remains essential. Strategic deployment of both optimal plant height and shorter duration will be crucial for developing new climate resilient, high yielding varieties to improve future productivity growth.

The realized genetic gains in rice grain yield documented across major global breeding programs underscore the substantial progress achieved through targeted selection and breeding strategies, while also highlighting persistent challenges in accelerating yield improvements under diverse environmental constraints. In our study, we estimated genetic gains of 0.34% in rainfed lowland, 0.27% in rainfed upland, and 0,14% in irrigated lowland ecology. These gains, while positive, remain far below the desired rate of ≥1.5% needed to meet rising consumption demands (Li et al., 2018). However, this trend is consistent with some recent findings of other rice breeding programs, where genetic gains often remain below 0.5%, though some programs have achieved gains exceeding 1% (Juma et al., 2021; Kumar et al., 2021; Khanna et al., 2022; Pereira de Castro et al., 2023; Rahman et al., 2023; Khanna et al., 2024; da Silva et al., 2025). As summarized in **Table 5**, absolute genetic gains ranged from low increments ranged from 0.1-0.46% under irrigated conditions and 0.68-1.9% under stress conditions such as drought in most of the major breeding programs. Though some programs such as Embrapa (Brazilian Agricultural Research Corporation) upland rice breeding program have achieved substantial gain of 3.08% per year. A meta-analysis of 29 studies from 1999 to 2023 found yield gains ranging from 0.1% to over 3.0% (Seck et al., 2023). Thus, results from this study align with most of the reports of low genetic gain trends but also underscore areas for improvement in grain yield, across the ecologies. Low genetic gain in some Asian breeding programs for favorable environments has been reported due to insufficient multi-location testing, low selection intensity, and prolonged breeding cycles due to the excessive use of older parental lines (Cobb et al., 2019; Rahman et al., 2023; Biswas et al., 2024). This is important to consider that compared with breeding programs in Asia (e.g., IRRI, BRRI) and Latin America (e.g., Embrapa), AfricaRice operates in more heterogeneous and stress-prone environments of sub-Saharan Africa, where multi-stress tolerance and broad adaptation take precedence over maximum yield potential. Consequently, genetic gains tend to be slower but more stable across environments. The findings from this study therefore complement global analyses by providing a regional perspective on the pace and direction of breeding progress under the unique constraints of African rice ecosystems.

**Table 5:** Reported realized genetic gains in rice grain yield from major breeding programs globally.

Region/ breeding program	Ecology	Germplasm studied	Period of release/evaluation	Absolute gain _grain yield (kg ha^−1^ yr^−1^)	Relative gain_ grain yield (% yr^−1^)	References
Asia/IRRI	Irrigated	Released varieties and breeding lines	1960-2014	8.75^*^ 17.36^#^	0.23^*^ 0.46^#^	Juma et al. (2021)
Asia/IRRI	Rainfed lowland (drought prone)	Breeding lines	2003-2019	2.29-9.52	0.13-0.55	Khanna et al. (2022)
Asia/BRRI	Irrigated	Released varieties	1970-2020	10-20	0.18-0.28	Rahman et al. (2023)
Asia/IRRI (Philippines and Bangladesh)	Irrigated (Salinity prone)	Released varieties and breeding lines	2000-2019	1.52-14.2^*^ 2.2-5.9^#^	0.1-0.31^*^ 0.12-0.14^#^	Khanna et al. (2024)
Asia/IRRI Drought Breeding Network -India	Rainfed lowland (drought prone)	Breeding lines	2005-2014	25-34	0.68-1.9	Kumar et al. (2021)
Brazil/Embrapa	Upland (favorable)	Breeding lines	–	67.8	3.08	Pereira de Castro et al. (2023)
USA/LSU	–	Breeding lines	1912-2022	4.55	0.07	da Silva et al. (2025)

BRRI, Bangladesh Rice Research Institute; IRRI, International Rice Research Institute; EMBRAPA, (Brazilian Agricultural Research Corporation); LSU.

^*^Breeding lines; ^#^released varieties.

The lower gain in rainfed environments particularly in upland reflects persistent constraints in this ecology, where soil fertility is often poor, drought and biotic stresses such as weeds and diseases are more pronounced in SSA. In rainfed lowland ecologies, lower grain yields are largely attributed to abiotic stresses such as flooding, drought, and iron toxicity (Saito et al., 2018; Van Oort, 2018; Saito et al., 2023). Furthermore, breeding progress in rainfed systems is often slower due to limited resources, number of high-yielding, climate-resilient varieties and the slow varietal turnover rate. Recent reports on varietal adoption in various SSA countries show that, as of 2008, only 40% of cultivated rice areas in 18 Sub-Saharan African countries were planted with improved varieties, many released before 2000 (Futakuchi and Saito, 2021). For instance, in rainfed upland ecology, NERICA varieties, particularly NERICA 4, still dominate farmer fields and exhibit broad adaptation traits, but their longevity as “mega varieties” may hinder genetic progress. Similarly, other older varieties remain widely grown, and their replacement has been a key objective and challenge of African breeding program. To enhance future gains, breeding programs in SSA must transitioning toward early maturing, high yielding, market-oriented and climate-resilient product pipelines, integrating trait discovery, farmer and consumer preferences, and effective seed delivery systems.

Global projections show that current rates of rice yield improvement (approximately 1.0% annually) fall short of the 2.4% needed to double production by 2050 (Ray et al., 2013). Addressing this genetic gain gap requires optimization of the breeder’s equation [*R_t_ = (h^2^×S)/t, (R_t_ = genetic gain per period of time, often in units/year; h^2^=narrow or broad sense heritability; S = selection differential in units; t = period or cycle, often in years*], particularly by reducing breeding cycle time (t) and increasing selection accuracy (Cobb et al., 2019; Baertschi et al., 2021; Dreisigacker et al., 2023). To address the low rate of genetic gain, AfricaRice breeding programs have adopted an elite x elite population improvement breeding strategy in all the breeding pipelines for prioritized market segments in Africa. Replacing pedigree breeding with the single-seed descent method coupled with rapid generation advancement (RGA) significantly reduced the breeding cycles and provided more than a 50% reduction in cycle time compared to the past (AfricaRice, 2023). In addition, AfricaRice has integrated marker-assisted selection via major allele selection and genomic selection (GS) with digitalized data capture in the breeding programs to increase selection accuracy and intensity by predicting genomic estimated breeding values (GEBVs) (Hassen et al., 2018; Ahmadi et al., 2020). Incorporating multi-environment trial designs and mixed-model analysis with multi-trait selection indices has further enhanced heritability and precision (Cooper et al., 2023; Nguyen et al., 2023). These advances are expected to further enhance genetic gains and reduce disease resistance breakdown through early recycling of superior lines (Khanna et al., 2024).

## 5 Conclusion

This study provides a comprehensive assessment of genetic trends in AfricaRice and partners-bred varieties across Sub-Saharan Africa’s (SSA) major rice ecologies, namely, irrigated lowland, rainfed lowland, and upland systems. The findings reveal low progress in grain yield improvement, with annual genetic gains varying from 0.14 to 0.34% (7–12 kg/ha). While these gains underscore the efficacy of breeding efforts, they fall short of the ≥1.5%/year required to meet SSA’s escalating rice demand, driven by population growth, urbanization, and climate-induced yield instability. To bridge this gap, a transformative approach integrating advanced breeding technologies with vibrant and robust seed dissemination systems is critical. Modernizing breeding programs through genomic selection and rapid generation advancement can significantly shorten breeding cycles and improve trait selection for climate resilience. Prioritizing key traits like drought, flood, and heat tolerance ensures that crops are better suited to changing environments. Equally important is strengthening varietal turnover by promoting farmer adoption through targeted dissemination, policy support for climate-smart seeds, and widespread awareness campaigns using digital tools to ensure that yield gains at the research level translate into improved productivity on farmers’ fields. In parallel, the adoption of standard agronomic practices and good agricultural practices (GAP) is essential to realize the full potential of genetic interventions under farmers’ field conditions. These integrated interventions are essentially required to achieve the targeted genetic gain of >1.5%/year in SSA.

## Data Availability

The original contributions presented in the study are included in the article/**Supplementary Material**. Further inquiries can be directed to the corresponding author.
